# Effects of maternal calcium propionate supplementation on offspring productivity and meat metabolomic profile in sheep

**DOI:** 10.1371/journal.pone.0294627

**Published:** 2023-12-20

**Authors:** Luis Fernando Pérez Segura, Rogelio Flores Ramirez, Alejandro E. Relling, José Alejandro Roque-Jimenez, Naifeng Zhang, Einar Vargas-Bello-Pérez, Héctor A. Lee-Rangel

**Affiliations:** 1 Facultad de Agronomía y Veterinaria—Centro de Biociencias Universidad Autónoma de San Luis Potosí, Soledad de Graciano Sánchez, San Luis Potosí, México; 2 CONACYT Research Fellow, Coordinación para la Innovación y Aplicación de la Ciencia y la Tecnología (CIACYT), San Luis Potosí, SLP, México; 3 Department of Animal Science, The Ohio State University, Ohio Agricultural Research and Development Center (OARDC), Wooster, OH, United States of America; 4 Institute of Feed Research of Chinese Academy of Agricultural Sciences, Key Laboratory of Feed Biotechnology of the Ministry of Agriculture and Rural Affairs, Beijing, China; 5 Department of Animal Sciences, School of Agriculture, Policy and Development, University of Reading, Reading, United Kingdom; 6 Facultad de Zootecnia y Ecología, Universidad Autónoma de Chihuahua, Chihuahua, Mexico; Tokat Gaziosmanpaşa University: Tokat Gaziosmanpasa Universitesi, TURKEY

## Abstract

This study determined the effect of dietary calcium propionate (CaPr) as a source of energy supplementation during the First Half of Gestation (FMG), the Second Half of Gestation (SMG), and during All Gestation (AG), on offspring post-weaning growth performance, meat quality, and meat metabolomic profile. Thirty-one pregnant ewes were assigned to one of four treatments: a) supplementation of 30 gd−1 of CaPr during the first half of gestation (day 1 to day 75, n = 8) (FMG); b) supplementation of 30 gd−1 of CaPr during the second half of gestation (day 76 to day 150, n = 8) (SMG); c) supplementation of 30 gd−1 of CaPr during all gestation (AG, n = 8); d) no CaPr supplementation (control; CS, n = 7). The ewes were ad libitum fed a basal diet based on oat hay and corn silage. Ewes were distributed in a completely randomized unbalanced design to four treatments. The FMG group had lower (P ≤ 0.05) birth weight and weaning weight than the CS group. However, the average daily gain was similar across all treatments. Empty body weight and FMG had lower values (P ≤ 0.05) than the other groups. Both FMG and AG had lower hot carcass weight (P ≤ 0.05) compared to CS, while CaPr treatments resulted in reduced hot carcass yield (P ≤ 0.05). Meat color and texture were similar among treatments. A principal component analysis between gestation stages showed a trend for separating CS and FMG from SMG and AG, and that was explained by 93.7% of the data variability (PC1 = 87.9% and PC2 = 5.8%). Regarding meat metabolomic profile, 23 compounds were positively correlated between all treatments. Only 2 were negatively correlated (eicosane and naphthalene 1,2,3); but tetradecanoic acid, hexadecane, undecane 5-methyl, (-)-alpha, hexadecenoic acid, octadecanoic acid, and octadecane had a highly significant correlation (P ≤ 0.05). Overall, dam supplementation with CaPr during different periods of gestation provoked changes in meat metabolites related to the biosynthesis of fatty acids in lambs without negative changes in lamb’s growth performance and carcass quality.

## Introduction

Since the 1950s, animal scientists have been studying the consequences of maternal nutrition during pregnancy on the growth and development of the offspring [1]. There is increasing interest in minimizing the consequences of environmental and management effects that could impair animal productivity and efficiency [2, 3]. Maternal nutrition could be considered as a major factor in the intrauterine environment, and this is known as fetal programming [4]. Furthermore, it has been established that maternal nutrition during different stages of pregnancy can induce permanent changes in the metabolism of the future offspring [5, 6].

The dam’s malnutrition during pregnancy could promote intrauterine fetal growth restriction and result in low birth weight [7, 8], and offspring born from these dams may have reduced daily weight gain [9] and inadequate development of skeletal muscle and adipose tissues [10]. Although restricted maternal nutrition has been reported as a factor that can increase fat deposition in the offspring of other livestock species [11, 12], the impact of maternal nutrition on offspring carcass traits in ruminants remains a research field that deserves more attention.

In recent years, research on muscle development and meat science has increased the use of metabolomic analysis [13, 14]. The metabolomic approach has been employed widely in many research fields of animal nutrition and physiology [15, 16] because of its high-throughput capacity. The compounds analyzed are the key metabolites such as flavor-associated compounds, nutrients, and functionality-associated compounds in food, some of which have a molecular weight of >1000 Da. Metabolomics analysis is a powerful tool for understanding the biologically and agriculturally meaningful information from global metabolome profiles and the changes caused by factors in food production processes [17].

Some studies have used a metabolomics approach to describe the effect of diet on growth, fattening phase, and meat quality [18, 19]. However, few studies have used metabolomics approaches in experiments that evaluate maternal nutrition and its effects on offspring in small ruminants [20]. Polizel et al. [21] reported that in ewes, during gestation, overfeeding or restricting feed could alter the offspring’s *Longissimus* muscle metabolome. As per the research conducted by Du et al. [2], the fetal environment plays a critical role in meat production, as it increases the number of muscle fibers in the offspring, which only happens during the fetal phase. Thus, maternal energy intake during early to mid-gestation decreases the number of myofibers in the offspring impairing the muscle mass development during the fattening phase [2]. In addition, the authors described that maternal nutrient deficiency demonstrated long-term effects on offspring productive performance.

Recently, the modulation of the dietary energy levels during rumen fermentation [22], is one of the strategies proposed for increasing the dietary energy intake during the gestational periodis. Zhang et al. [23] have suggested that calcium propionate (CaPr) can play an active role in overcoming problems related to energy balance during gestation and reduce the risk of ketosis and hypocalcemia during lactation in dairy cows. At the same time, different studies have concluded that CaPr dietary supplementation in ruminants could increase the concentration of propionate in the rumen, boosting glucose synthesis in the liver [24, 25]. Despite the importance of dietary energy intake during fetal development [23]. There is scarce data on the use of energy promoters such as CaPr on different gestation periods in ruminants.

Therefore, the current study hypothesized that CaPr supplementation in gestation ewes in different periods increases the offspring’s post-weaning performance in growth, development, meat quality, and meat metabolomic profile. Thus, this study aimed to determine the effects of dietary CaPr supplementation of ewes during the First Half of Gestation (FMG), the Second Half of Gestation, and during All Gestation (AG) on offspring post-weaning growth performance, meat quality, and meat metabolomic profile.

## Materials and methods

### Ethics

The animal procedures were reviewed and approved by the Committee for the Ethical Use of Animals in Experiments of the Universidad Autónoma de San Luis Potosi (project #807944), according to the regulations and standards that are required by the Mexican government for the use of animals for several diverse activities. Federal law on technical specifications for the care and use of laboratory animals and for livestock farms; centers of production, reproduction, and breeding; zoos; and exhibition halls must meet the basic principles of animal welfare (NOM‐062‐ZOO‐1995).

### Animals, experimental design, and treatments

The study was conducted at the sheep experimental facilities of the Facultad de Agronomía y Veterinaria of Universidad Autónoma de San Luis Potosí, Soledad de Graciano Sánchez, San Luis Potosí, México (Latitude 22°14′0.58″; Longitude 100°50′48.5″).

**Fig 1:**
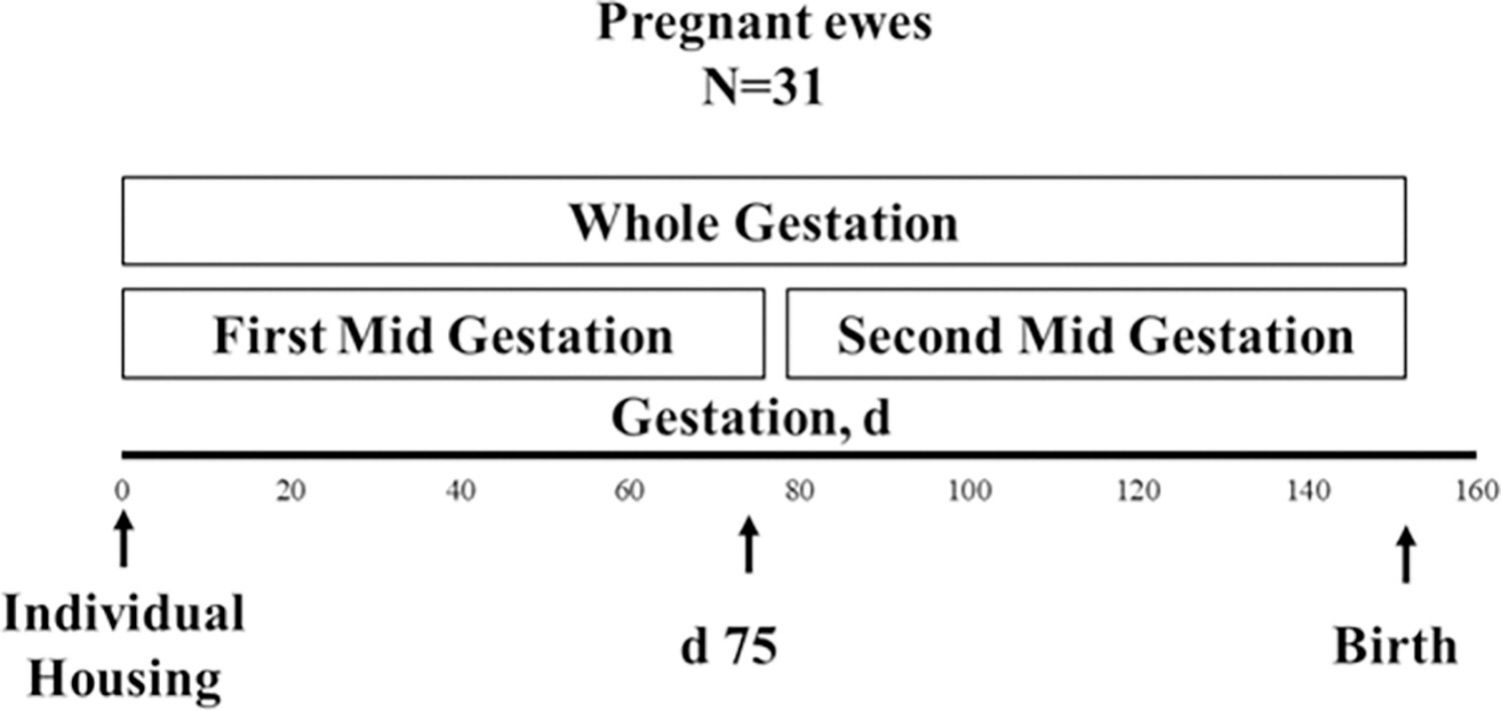
Experimental design to evaluate the effects of CaPr supplementation on offspring meat characteristics and metabolomics analysis.

Thirty-one (2 to 3-year-old) Rambouillet ewes (initial body weight (IBW) 47.2 kg ± 4.8) were randomly assigned to one of four treatments. Ewes were all mated by one Rambouillet ram (IBW 72.4 kg). The ram used a marking harness with paint during breeding. Immediately after mating, ewes were housed in individual roofed pens (3 × 4 m^2^) with straw bedding and a device to provide free access to water. The day of the mating was considered as day one of conception. Thirty-five days after mating, ultrasonography was used to confirm pregnancy. Afterward, by using a completely randomized design, 31 pregnant ewes were assigned to one of four treatments: a) supplementation of 30 gd−1 of CaPr during the first half of gestation (day 1 to day 75) (FMG); b) supplementation of 30 gd−1 of CaPr during the second half of gestation (day 76 to day 150) (SMG); c) supplementation of 30 gd−1 of CaPr during the whole gestation (AG); d) no CaPr supplementation (control; CS) (Fig 1). The dose of CaPr supplementation was based on a previous study on lambs during the fattening phase (24,25), in which supplementation at a similar dose had positive effects on mRNA abundance on hypothalamic gene expression and lambs’ growth. The CaPr (Nutryplus, Queretaro, Mexico) was mixed with concentrate using a feeding wagon each day.

Regardless of treatment, every day at 1200h, ewes were fed 300 gd^-1^ of concentrate (10% of CaPr). The ewes were fed (0800 h) *ad libitum*, a mix 50:50 of oat hay and corn silage. The forage mix had 47% dry matter, 14% acid detergent fiber, 29% neutral detergent fiber, 14% crude protein, and 4% ether extract. Diets followed the recommendations for nutrient requirements for sheep during each physiological stage (early and mid-gestation and lactation; NRC, [26]). At weaning, forty-three lambs were obtained (initial body weight (IBW) 4.4 ± 1.1 kg). At day 60, five lambs (two females and three males) were discarded due to their small size and slow growth, the slow development could be because some of these animals had respiratory and diarrhea problems during the study, resulting in 12 lambs from FMG, 10 lambs from SMG (two discarded), 12 lambs from AG, and 9 lambs from the CS group (three discarded). All lambs were fed exclusively on maternal milk until weaning.

Feed samples were collected weekly, ground, and analyzed according to the AOAC [27] for dry matter ((DM, method number 981.10)), crude protein (Nitrogen × 6.25; CP, method number 967.03), ether extract (EE, method number 920.39), crude fiber (CF, number method 978.10), and ash (method number 942.05).

### Offspring performance

The study lasted 60 days. The lambs were weighed at birth and weaning (60 days later). The average daily gain was calculated during this period. After weaning, all lambs were sent to the Municipal slaughterhouse of San Luis Potosi. Before slaughter, shrunk body weight (SBW) was recorded after feed and water were withdrawn for 24 h. Lambs were slaughtered humanely following the Mexican Official Norms [NOM-08-ZOO, NOM-09-ZOO, and NOM-033-ZOO] established for the slaughtering and processing of meat-producing animals. Immediately after slaughter, carcass weight was recorded, and the components not included in the carcass (trachea, lungs, heart, liver, spleen; full and empty digestive tract, head, skin, and feet apart from the testicles) were weighed for individual carcass yield calculations [28]. The *Longissimus dorsi* of each carcass was divided from the 6^th^ until the 13^th^ rib and individual samples were packed under a high vacuum and frozen at -20°C for subsequent analysis.

#### Meat characteristics

Konica Minolta on Color CM-2500 is an instrument used to measure meat color based on L* a* and b* parameters. Whereby, the L* value represents the light to dark color, a* represents green to red color, and b* represents the blue and yellow tones. The equipment was calibrated with a blank before each measurement. For texture measurements, raw meat toughness was determined in samples of 1×1×3 cm, using a compression test, and this was carried out at room temperature (20 ± 2°C), applying up to 20% strain at a speed of 50 mm/min using an Instron Universal testing machine, model 3365 (Instron Corporation, High Wycombe, UK), equipped with a modified compression cell that prevents transverse elongation of raw meat. Five determinations were obtained from each sample.

#### Volatile organic compound’s fingerprints

For the determination of the chemical footprint of the meat, Cyranose® 320 (Sensigent, California, US) was used. This equipment has a portable electronic nose that has 32 carbon polymer-composite chemoresistors incorporated into a matrix that adsorbs the volatile organic compounds (VOCs) from the meat samples causing an increase in the electrical resistance of each sensor. Each chemoresistor has different properties in the adsorption of VOCs producing different degrees of response.

Briefly, two grams of meat from each lamb were placed in a 20 mL vial sealed with a silicone cap (Agilent® 75.5 × 22.5, CA, USA). Each vial was incubated at 60°C for 15 minutes in a heating sand bath. Later, the needle of a vacuum pump (Millipore XX5411560, Burlington, MA, USA) punctured the silicone cap of the vials to supply the VOCs from meat to three different lines. The first line supplied nitrogen gas, while the second line contained the VOCs from the meat sample. This gas mix was distributed to a Tedlar bag by the third line. Finally, the configuration of the electronic nose consisted of a constant flow of 120 mL/min during 40 seconds of baseline recording with ultrapure nitrogen passing through the extraction system to remove the noise caused, and a 46-second period of sample analysis, subsequently increased to a flow of 180 mL/min of ultrapure nitrogen for the purging of the sample line and air intake, with a substrate temperature of 32°C. During the analysis, the instrument recorded the increase in electrical resistance of each sensor resulting from the adsorption of VOCs onto the sensors.

#### GC-MS meat analysis

The extraction of meat compounds was performed using an ultrasonic processor (GEX130, 115 V 50/60 Hz) equipped with a 3 mm titanium tip and mechanical stirrers (Cole-Parmer, IL, USA). One gram of meat was mixed with 10 mL of hexane. Subsequently, the organic phase was separated, concentrated to 1 mL of extracted mixture, and evaporated (Zymark, Turbovap LV Concentration Evapotarot, NB, USA) for the final analysis.

The characterization of meat was performed with a gas chromatograph (GC-HP 6890) coupled with mass spectrophotometry (MSHP 5973), equipped with a capillary column 60 m length, 0.255 mm diameter, and 0.25 μm film thickness (HP 5MS, Agilent). The temperature program was 70°C for 2 min, which was then increased to 250°C at the rate of 20°C/min, then to 290°C at the rate of 5°C/min, then increased to 300°C at the rate of 1°C/min, then to 310°C at the rate of 5°C/min and kept there for 36 min. The injector temperature was 250°C in spitless mode. The helium flow rate was 1 mL/min. The mass spectrophotometry was programmed in SCAN mode (50–500 m/z) to identify compounds.

### Statistical analysis

The experimental design was a completely randomized unbalanced design with four treatments. The type of birth (single or double) was used as a covariate to account for any unwanted variation within the treatment group. The SAS [29] MIXED procedure was used to analyze data; the lamb was the random variable, the mother supplementation was fixed, and the lamb nested in treatment was residual. Means comparison was performed with the LSMEANS procedure and the ADJUST Tukey option [29]. A P-value of 0.05 was selected as the significance level.

For the aromatic footprint, the multivariate analyses were performed using the increase in resistance of the 32 sensors obtained from the fractional difference: ΔR/Ro = (Rmax-Ro)/Ro where R is the maximum system response of each sensor, and Ro is the reference reading of each sensor with ultra-pure nitrogen.

The multivariate statistical analysis of meat metabolites was performed using the metabolomics data processing tool, MetaboAnalyst 5.0. The metabolite data were transformed using the generalized log transformation and then range-scaled to correct for heteroskedasticity and to reduce mask effects. Partial least squares discriminant analysis (PLS-DA) and variable importance in projection (VIP) were performed using R to identify the differential metabolites among groups and to rank the metabolites according to their importance in discriminating groups.

## Results

### Growth trial, carcass quality, and meat characteristics

Performance in post-weaning lambs is shown in Table 1. Differences in weight were detected. Lambs from FMG treatment were lighter in birth weights (P ≤ 0.05) than those from other treatments. Lambs from FMG were lighter (P ≤ 0.05) at weaning than the other groups. The average daily gain was similar among groups.

**Table 1:** Effects of maternal supplementation with 30gd^-1^ of CaPr in the first half of pregnancy (FMG), the last half of pregnancy (SMG), all gestation (AG), and not supplementation (CS) on performance in post-weaning lambs at 60 days of age.

	CS	FMG	SMG	AG	SEM
**Birth weight, kg**	5.12^b^	3.93^a^	4.77^a^^b^	4.82^a^^b^	0.27
**Weaning weight, kg**	19.23^b^	16.88^a^	18.11^a^^b^	18.10^a^^b^	1.27
**Difference, kg**	14.11^b^	12.95^a^	13.33^a^	13.15^a^	1.15
**Average Daily Gain, kg**	0.23	0.21	0.22	0.21	0.01

^a,b,c^ Means within a row with different superscripts differ (P < 0.05); SEM, standard error of the mean.

Empty body weight was lower (P ≤ 0.05) in FMG, hot carcass weight was lower (P ≤ 0.05) in both FMG and AG, while hot carcass yield was reduced (P ≤ 0.05) with CaPr treatments (FMG, SMG, and AG) (Table 2). The meat color was similar between treatments. The CS treatment showed the lees values for texture compared to all the CaPr treatments, reflected in the tenderness of the meat (P < 0.05).

**Table 2:** Carcass and meat quality at slaughter post-weaning lambs from ewes supplemented with 30gd^-1^ of CaPr in the first half of pregnancy (FMG), the last half of pregnancy (SMG), all gestation (AG), and not supplementation (CS).

Item	CS	FMG	SMG	AG	SEM
**Carcass quality**					
**Empty body weight, kg**	18.04^b^	16.80^a^	18.48^b^	17.87^b^	1.26
**Hot carcass weight, kg**	10.04^b^	8.32^a^	9.29^a^^b^	8.53^a^	0.91
**Hot carcass yield, %**	55.90^b^	49.54^a^	49.81^a^	47.73^a^	2.85
**Meat characteristics**					
L*	39.5	39.5	39.5	39.5	0.27
a*	14.4	14.8	15.8	16.2	0.08
b*	7.41	7.14	7.57	7.36	0.35
C*	14.9	14.4	15.2	14.8	0.26
**Texture, N/cm** ^**2**^	23.2^a^	39.5^b^	36.4^b^	36.5^b^	2.39

^a,b,c^Means within a row with different superscripts differ (*P* < 0.05); SEM, standard error of the mean; L* represents the light to dark color, a* represents green to red, and b* represents the blue and yellow tones.

### PCA of volatile organic compounds fingerprints

**Fig 2:**
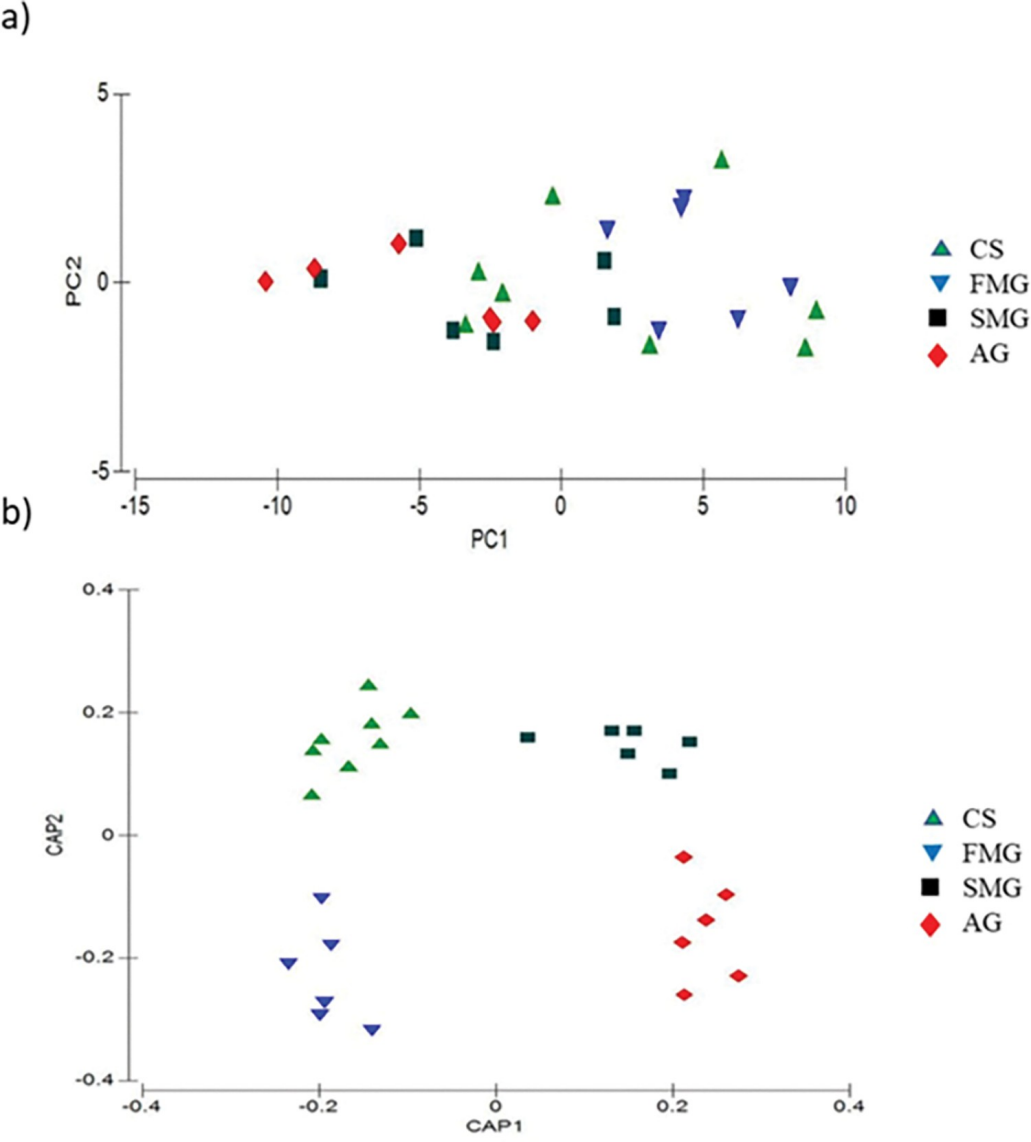
**a)** PCA-loading map with the changes in volatile organic compounds fingerprints in meat lamb from ewes supplemented with CaPr during gestation; **b)** Discriminant analysis map determined by discriminant factors (treatments) for volatile CS, FMG, SMG, and AG.

A principal component analysis between gestation stages showed a trend for separating CS and FMG from SMG and AG, explaining 93.7% of the data variability (PC1 = 87.9% and PC2 = 5.8%) (Fig 1). With a canonical discriminant analysis model, a separation was observed between the chemical fingerprints of CS and FMG for SMG and AG on the first canonical axis CAP1 with a correlation of r2 = 0.9788 from detected VOCs. Likewise, a separation was observed across the CAP2 axis from CS and SMG and AG and FMG, and the overall correct classification of chemical fingerprints of 77.77% (P = 0.0001) (Fig 2).

### Metabolomic meat analysis

**Fig 3:**
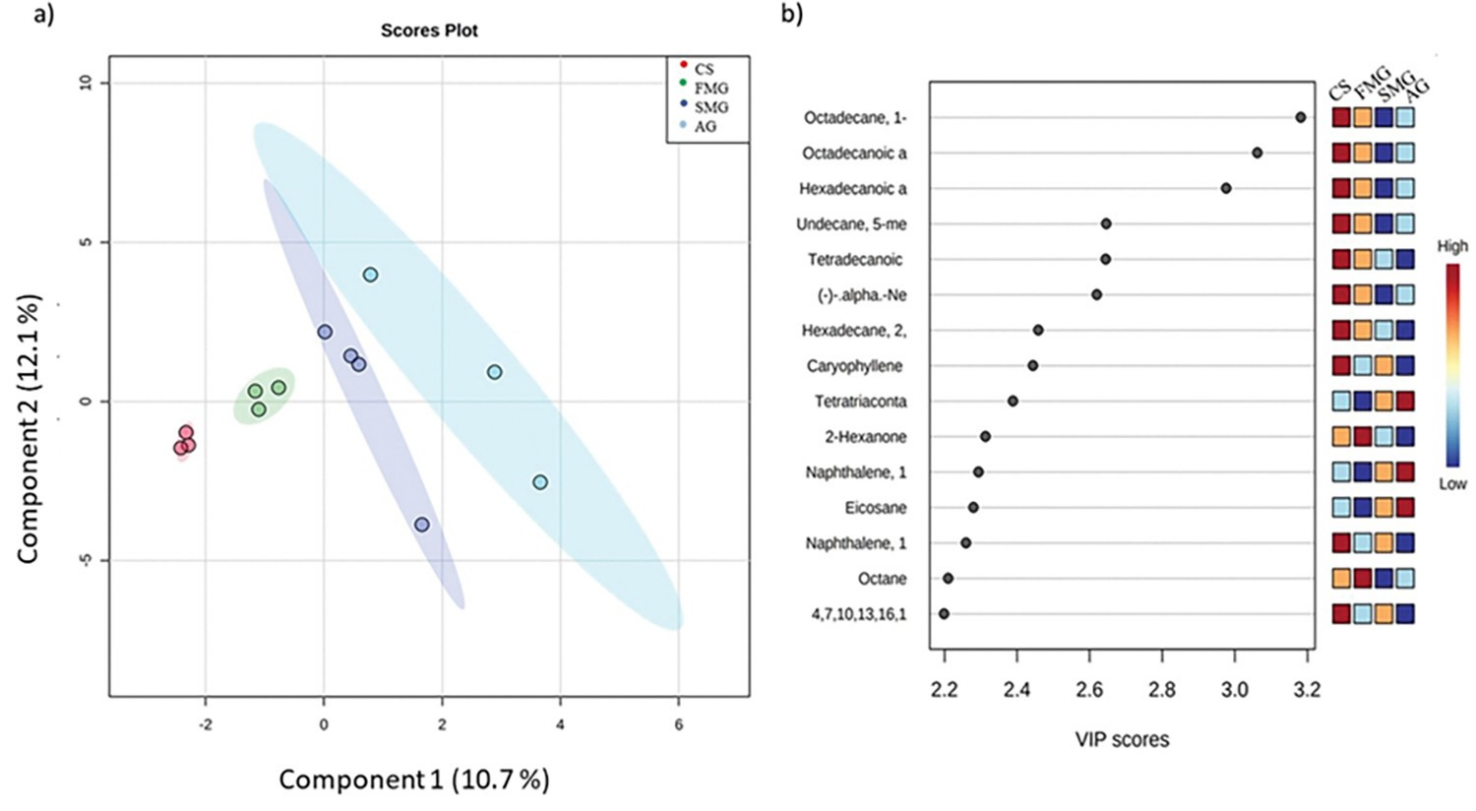
a) Partial least squares discriminant analysis (PLS-DA) score plot in 2D graphs using the concentration of all metabolites quantified by groups. b) Variable importance in projection (VIP) plot analyses obtained from meat lambs.

To establish the metabolomics profiles of the treatments, which showed differences (Fig 3A), using PLS-DA, pairwise comparisons were conducted to elucidate the specific differences between groups. Twelve samples were classified as treatment and analyzed using MetaboAnalyst, which resulted in a maximum separation of meat samples, which accounted for 12.1% (maximum) variation from the dataset. The top 15 metabolites discriminating CS, FMG, SMG, and AG are shown in Fig 3B. A higher VIP value was associated with a CS. According to the VIP score, the most important metabolites in differentiating treatments were octadecane, octadecanoic, hexadecenoic, undecane, tetradecanoic, (-)-alpha, hexadecane, caryophyllene, tetratiaconta, 2-hexanone, naphthalene, eicosane, naphthalene-1, and octane.

**Fig 4:**
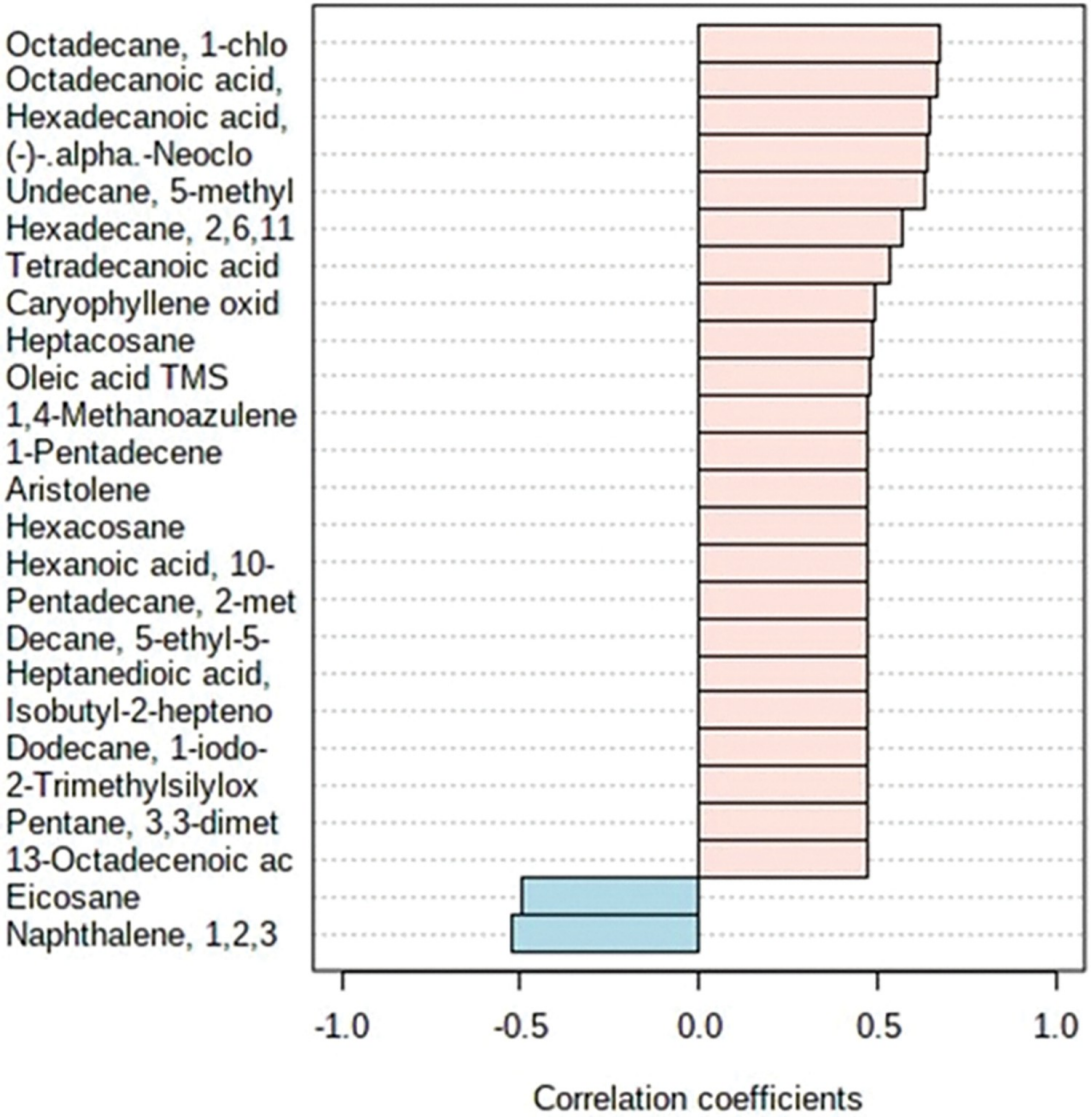
The loading plot reports the correlation between the 25 most important compounds between treatments. Highly significant correlations (*P* ≤ 0.05) are up.

Twenty-three compounds were positively correlated between all treatments; only two were negatively correlated (eicosane and naphthalene 1,2,3); but tetradecanoic acid, hexadecane, undecane 5-methyl, (-)-alpha, hexadecenoic acid, octadecanoic acid, and octadecane showed a significant correlation (P ≤ 0.05) (Fig 4).

## Discussion

It is known that maternal conditions influence the fetus’s development and later affect its productive performance [10]. However, these results could be an outcome of postnatal factors, which affect growth performance throughout the lambs’ life [30]. As far as we know, there is limited data on how CaPr supplementation affects ruminants during gestation with concomitant effects on the lamb’s performance. It is worth pointing out that in this study, the baseline diets were formulated to contain equal amounts of CaPr for all treatments.

Since the 1940s, it was suggested that the fetus is affected by the dam’s nutrition [31]. Later, during the second world war in humans, Baker et al. [32] concluded that maternal nutrient restriction negatively affects the offspring and increases the risk of multiple diseases, principally metabolic-related problems. Recently, maternal nutrition has been implicated in postnatal changes that could impact long-term offspring health and performance [30, 33]. Vonnahme et al. [34] revealed that restricted maternal nutrition could negatively impact fetal development. Maternal nutrition impact on lamb birthweights depends on the degree of over or under-feeding and gestation stage [30]. Maternal nutrition during late gestation has great consequences on birthweight [35]. Nevertheless, early, and mid-gestation nutrient levels have additive effects on postnatal growth and adult performance [36]. Thus, it can be considered that postnatal growth could be affected by alterations in early- and mid-gestation maternal nutrition, combined with the prenatal environment [37].

Placental development occurs during the first and beginning of the second third of gestation [38], with a maximum development between 40 and 60 days of gestation [39]. Maternal nutrition reduces angiogenesis and could influence placental development [40]. In the case of maternal nutrition, blood flow between the uterus and placenta is compromised, negatively affecting the nutrient supply to the fetus, causing a reduction in fetal development [41]. Although placental development occurs early in pregnancy, developmental delays could affect fetal development during the last third of gestation and at birth [34].

Our study shows contrasting findings to previous studies where different sources of supplementation during gestation improved the offspring development [42, 43], given that in our study, the CS treatment showed heavier weights at birth and offspring development compared to the lambs that received supplementation with CaPr in different stages of gestation. Larson et al. [42] described that in some cases where there are no apparent fetal performance responses to maternal nutrition, the offspring might express a remodeling at molecular levels, such as epigenetic modifications or modulation in mRNA gene expression. According to Robinson et al. [1], the weight of a lamb at birth can be influenced by maternal nutrition by up to 20%. However, the mother’s age also plays a crucial role, accounting for a 5.5% impact. In our study, we employed multiparous ewes, and this factor may have interacted with the treatments, leading to increase weights from the control group. We estimated that calcium propionate supplied 3.96 Mcal/kg of DM [44], and this was enough to increase the available energy for the ewe during pregnancy, which may be used for fetus development and milk production and, consequently, impacted the lamb’s weaning weight. Thus, we may assume that the effect of maternal nutrition is extended until the lactation period, which allows better lamb nutrition and that should have influenced weight at weaning and slaughter of lambs from ewes supplemented with CaPr.

Lambs from FMG treatment had a lower empty body weight. Interestingly, Brochine et al. [45] used chromium propionate and calcium salts during gestation, and they reported that the weight of non-carcass components of lambs was heavier than control groups. Also, the authors reported that chromium propionate during maternal supplementations affects lambs’ respiratory tract and spleen development. One of the spleen’s functions is synthesizing immune system cells and filtering the body’s red cells stimulating the production of immune cells and this results in healthier [43, 46]. However, in our study, the gastrointestinal tract was not characterized. Our result could be related to a greater gastrointestinal weight improvement by CaPr as observed in the carcass performance. Future studies should explore the effect of using CaPr on a lamb’s immune system and gastrointestinal development.

In lambs, undernutrition could affect performance at weaning, provoking metabolic remodeling to ‘save’ energy, and this can result in greater fat deposition and lesser muscle mass [47]. These phenomena respond to mesenchymal stem cell (MSC) proliferation that originates in muscle and adipose tissues by transcription factors [2]. The main transcription factor for MSC differentiation is Wingless and Int (Wnt) signaling. Du et al. [2] mentioned that the Wnt signaling pathway could increase myogenesis and reduces adipogenesis in skeletal muscle, regulating body fat and reducing obesity susceptibility during fetus development. Additionally, Pan et al. [48] reported a genome identification and described that the Wnt family genes in cattle affect adipocyte differentiation in muscle. Thus, adequate levels of nutrients during gestation will increase Wnt signaling encouraging more myogenesis in early and mid-gestation [2, 49]. Therefore, at the end of gestation, adipogenesis increases, and myogenesis is inhibited due to a lack of signaling of Wnt [48].

Regarding carcass parameters, the role of osseous tissue is important as bone cells have many mitochondria, which supports the premise that the formation and remodeling of osseous tissue require great amounts of energy [2]. The bone tissue is highly vascularized, facilitating the exchange of nutrients and minerals. This process regulates calcium and plasma phosphorus levels through the influence of hormones, such as parathyroid hormone (PTH), calcitonin, and vitamin D [49]. Bone cells and osteoclast act on bone resorption and have many mitochondria, supporting the idea that energy utilization is essential in bone remodeling [46]. Also, mineral component modification of bone tissue improves the texture and meat quality regarding drip and thawing loss reduction. This idea supports the hypothesis that ewe diets with more energy can produce offspring with bones with a suitable mineral density that might improve the texture of meat quality, as observed in our study.

In meat quality, color and tenderness are the two most important characteristics. Some factors affect these characteristics, but the supply of vitamin E and fatty acids (FA) in the maternal diet is important to enhance them as FA supplementation improves α-tocopherol activity in the muscle [50]. Therefore, FA are important meat compounds responsible for sensory characteristics. Our data showed that feeding ewes with CaPr during different stages of gestation did not affect the lamb’s meat color. Nevertheless, texture characteristics were improved in raw meat from lambs supplemented with CaPr. The texture is important for the consumer’s acceptability and quality of meat products [51].

Rather than shear force, the compression test is correlated to the percentage of connective tissue responsible for lamb meat’s background toughness [52]. However, this is not a negative parameter, given that increased compression on fresh meat is related to improved tenderness [53]. According to Bhatt et al. [54], diets with high energy fed to Malpura cull ewes resulted in increasing marbling and new collagen synthesis. Thus, our results might be related to fetal development when fetuses receive greater amounts of energy from their mothers, increasing the intramuscular adipocytes and provided the sites for intramuscular fat, and increased intramuscular adipogenesis and collagen accumulation [51, 55].

Regarding lipid metabolism, the physiological stage of animals, influences around 90% of FA in cells originating from *de novo* synthesis [56], and this event occurs in the liver, where glucose is converted to pyruvate and then to citrate, and acetyl-CoA carboxylase (ACC) and malonyl-CoA functions as intermediates in FA synthesis and as regulators that control FA oxidation in liver and muscle as they regulate the entry of FA into mitochondria [57]. We observed a metabolomic change in the biosynthesis of FA, specifically on branched fatty acids analysis such as octadecane, octadecanoic (stearic acid C:18), and hexadecenoic (Palmitic acid C:16), with a higher correlation for the treatments. Those changes could indicate changes in the metabolism of the muscle or adipocyte cells in the muscle and the result may be a change in the expression of genes associated with FA metabolism. However, Coleman et al. [58] did not report differences in these genes in adipose tissue (LPL = lipoprotein lipase; ATGL = adipose triglyceride lipase; HSL = hormone-sensitive lipase; ELOVL2 = elongation of very long chain fatty acid 2; ELOVL4 = elongation of very long chain fatty acid 4; ELOVL5 = elongation of very long chain fatty acid 5; FABP4 = fatty acid binding protein 4; FAS = fatty acid synthase; FATP1 = fatty acid transport protein 1). Lambs from ewes in maternal nutrient-restricted diets reported a higher hepatic accumulation of triglycerides associated with lower gene expression of PPARγ [59]. This transcriptional regulator modulates adipocyte differentiation and fat metabolism. Similar to our results, the meat showed changes in the biosynthesis of fatty acids modulated by PPARγ since this transcription factor has been related to reducing lipid accumulation in muscle [60].

## Conclusion

Our study shows that maternal supplementation with CaPr positively impacts offspring productivity. Additionally, the nutritional management of dams supplemented with CaPr improves the offspring’s raw meat texture and the lambs’ meat metabolomic profile (more unsaturated fatty acids). Supplementing ewes with CaPr during gestation is an effective strategy that improves lambs meat. Further sensory and chemical analyses on lamb meat are recommended for future study.
